# Multi-sensory training and wrist fractures: a randomized, controlled trial

**DOI:** 10.1007/s40520-019-01143-4

**Published:** 2019-02-11

**Authors:** Bergthora Baldursdottir, Susan L. Whitney, Alfons Ramel, Palmi V. Jonsson, Brynjolfur Mogensen, Hannes Petersen, Ella K. Kristinsdottir

**Affiliations:** 1 Faculty of Medicine, University of Iceland, Reykjavik, Iceland; 2 The Icelandic Gerontological Research Institute, Landspitali University Hospital, Reykjavik, Iceland; 3 Department of Physical Therapy, Landspitali University Hospital, Tungata 26, 101 Reykjavik, Iceland; 4 Department of Physical Therapy, University of Pittsburgh, Pittsburgh, USA; 5 Faculty of Food Science and Nutrition, University of Iceland, Reykjavik, Iceland; 6 Akureyri Hospital, Akureyri, Iceland

**Keywords:** Fracture, Rehabilitation, Wrist, Sensation, Exercise

## Abstract

**Background:**

Asymmetric vestibular function, decreased plantar sensation, postural control and functional ability have been associated with fall-related wrist fractures.

**Objective:**

To investigate whether multi-sensory training (MST) improves postural control, vestibular function, foot sensation and functional ability among people with fall-related wrist fractures compared to wrist stabilization training (WT).

**Methods:**

This was an assessor-blinded, randomized controlled trial. Ninety-eight participants, age 50–75 years, were randomized to MST or WT. Pre- and post-training measurements: Head Shake Test (HST), Video-Head Impulse Test (vHIT), Semmes–Weinstein Monofilaments (SWF), Biothesiometer (BT), Sensory Organization Test (SOT), 10-m Walk Test (10MWT), Five Times Sit to Stand Test (FTSTS), Activities-Specific Balance Confidence (ABC) and Dizziness Handicap Inventory Scales (DHI). The training period was 12 weeks, with six supervised sessions by a physical therapist and daily home exercises for both groups.

**Results:**

There were significant endpoint differences in SOT (*p* = 0.01) between the two groups, in favor of the MST group, but no changes were seen in other outcome variables. Subgroup analysis with participants below normal baseline SOT composite scores indicated that the MST was more effective in improving 10MWT fast (*p* = 0.04), FTSTS (*p* = 0.04), SWF (*p* = 0.04) and SOT scores (*p* = 0.04) than the WT.

**Conclusions:**

MST improves postural control among people with a fall-related wrist fracture. The results further suggest that the program is more effective for those with SOT balance scores below age-related norms.

## Introduction

Postural instability and falls are one of the major health concerns associated with increasing age. About one-third of people aged 65 and over fall each year and the incidence of falls doubles every 5 years thereafter [1]. Injuries and fractures are common consequences of falls. Wrist fracture (distal forearm fracture) has been reported as the most common injury in people between 65 and 74 years of age, attending an orthopedic emergency clinic after a fall [2]. Wrist fractures have also been shown to be a strong predictor of future fracture risk [3] and are often a precursor to hip fractures [4], which results in increased health costs, decreased quality of life and even death [5].

The most common profile of a patients affected by a wrist fracture is a functionally independent woman younger than 75 years of age [6]. Although the majority of wrist fracture subjects are apparently healthy, many of them exhibit risk factors for new falls and fractures. These can be regular medications [7], functional decline [6], history of previous falls and fractures [7], as well as asymmetric vestibular function [8, 9]. In a recently published case–control study, postural control, plantar sensation, vestibular and physical functions were significantly worse among subjects having sustained a wrist fracture than healthy controls [10]. Furthermore, asymmetric vestibular function and decreased plantar pressure sensation showed the strongest associations with a fall-related wrist fracture [10]. Some of these variables, such as vestibular function, postural control and physical function, can be enhanced by rehabilitation.

Group sessions with vestibular rehabilitation have reduced the incident of vestibular asymmetry among elderly people with wrist fracture [11]. Vestibular rehabilitation consists of balance exercises and the incorporation of head movements that may provoke dizziness. Symptoms are generated by using exercises comprising a sequence of eye, head and body movements of increasing difficulty [12, 13]. Multi-sensory exercises are characteristically defined as exercises that selectively stimulate and manipulate all the three afferent sensory systems including vestibular, visual and somatosensory pathways [14–18]. Hu and Wollacott, reported that multi-sensory balance training designed to improve intersensory interaction, improved balance performance in healthy older adults [14] and optimized the muscle and movement characteristics among the participants [15]. Multi-sensory training directed at improving function of the sensory systems has improved functional mobility [16] and reduced body sway in older adults living in the community [17]. A pilot study on the efficacy of a new multi-sensory balance training, “The Reykjavik model”, consisting of combined mechano- and proprioceptive, vestibular and fall-prevention training, demonstrated that post-training, postural control, functional ability and confidence during daily activities improved among frail old people with a history of multiple falls [18].

Ongoing problems after a wrist fracture can encompass stiffness, pain and muscle weakness, which can lead to difficulties completing everyday functional tasks [19]. Physiotherapy usually consists of exercises for range of motion (ROM) and muscle strength to improve pain, range of motion, grip strength and activity in this population [20]. In the current emergency care settings in Iceland, people who have sustained a fall-related wrist fracture receive treatment for the fracture. When the cast has been removed (~ 5–6 weeks post-fracture), they are offered to participate in group training sessions with the aim to improve movement and strength in the wrist. Patients are referred to individual physical therapy sessions if their condition (range of motion restrictions and/or complex regional pain syndrome) requires further intervention. However, they are not routinely screened for falls and fracture risk nor offered an exercise program to improve or maintain balance control to reduce the risk of future falls.

The aim of the present study was to investigate whether, multi-sensory training (MST) “The Reykjavik model” improves postural control, vestibular function, foot sensation and functional ability among people with fall-related wrist fractures compared with those receiving wrist stabilization training (WT). Additionally, we wanted to investigate whether potential changes are affected by baseline balance control.

## Methods

### Design overview

This study was a randomized, controlled trial. All participants underwent baseline measurements within a week before intervention started. They were randomly assigned to one of two study arms: (1) intervention group = MST, and (2) control group = WT. Participants in both groups attended six treatment sessions (30 min each) during a 3-month period, which were supervised by a physical therapist (PT). In the first two training sessions, additional 30 min were allocated to the participants in both groups, so they could familiarize themselves with the proposed exercises. Participants in both groups further received a written exercise program that was to be performed daily at home. Duration of home exercises was a minimum of 15 min, without upper time limits in both groups. The participants kept a home exercise diary during the training period. Outcome measurements were repeated within a week after the last training session.

### Setting and participants

**Fig. 1:**
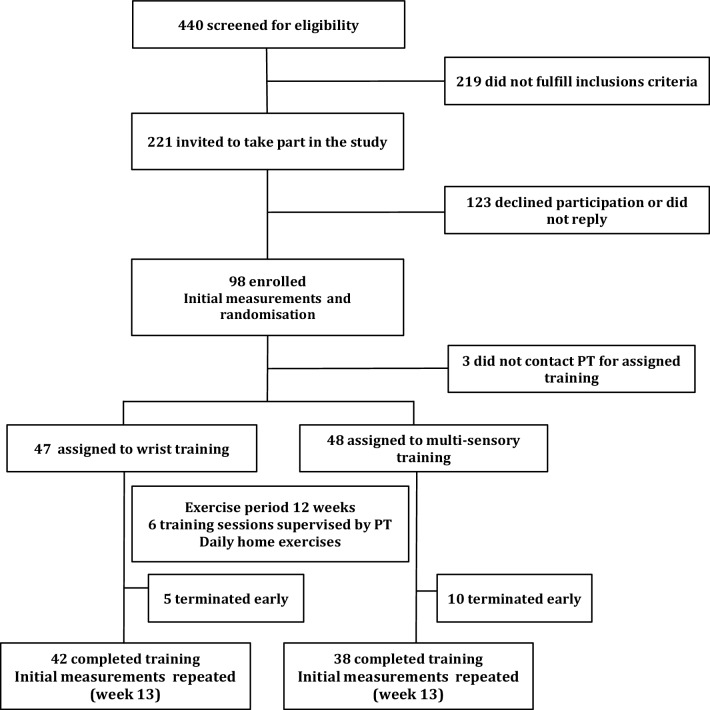
Study flow chart

Ninety-eight individuals (mean age 61.9 ± 7.1; range 50–75; females = 85, males = 13), who had previously sustained a fall-related wrist fracture, participated in this trial. They were identified from medical records at the Emergency Department of the Landspitali University Hospital in Reykjavik, Iceland and screened for eligibility from a total of 440 consecutive patients during a 12-month period. Enrolment commenced in May 2015 and ceased in May 2016. Subjects were recruited for the study 2–5 months after the fracture by an invitation letter, followed up by a phone call. Exclusion criteria were diagnosis of a degenerative CNS disease, such as Parkinson’s, Alzheimer’s or other diseases possibly impairing mobility or cognitive function. The study received permission from the Icelandic National Bioethics Committee (VSNb2013110036/03.11) and was conducted in accordance with the ethical standards laid down in the 1964 Declaration of Helsinki and its later amendments. The subjects provided informed written consent before participation in the study. A flow chart of study participants is shown in Fig. 1.

### Sample size

The recruitment target was 90 participants. Sample size calculations were based on previous work by Herbert et al. [21], where they used composite scores from the sensory organization test as the primary outcome measure. With a power of 80% and significance level of 5%, 36 people were required in each group in order to detect a mean difference of 10 SOT composite score assuming a standard deviation of 15. The sample size was increased to 47 participants per group to allow for a 30% loss due to dropouts.

### Randomization and interventions

After recruitment and baseline measurements, participants were randomized using a computer-created random number list and sealed envelopes.

### Intervention: multi-sensory training (MST), “the Reykjavik model”

The exercises were performed barefoot on firm and soft surfaces, during quiet stance and movements. Throughout all the exercises, the subjects’ attention was directed at weight distribution on the soles of the feet to recognize and control the position and movements of the body. They were also encouraged to be aware of their postural control pattern and taught to readjust their posture with slow smooth corrective motions at the ankles and avoid using high-frequency movements at the hips and upper body. The participants were discouraged to use their hands or body for external support. Control of posture was practiced during head movements in different directions, with eyes open, closed and during fixation of gaze. The subjects were taught how to react to sudden balance disturbances by taking a step to hinder falling and use stepping reactions when their stability was challenged by a manual push in different directions. Exercises were chosen from a list (Appendix 1) according to the main balance weaknesses of each subject, tailored close to the limits of capability, and progressed in the supervised sessions according to their improvements. The participants were instructed to perform the home exercises as well in barefoot, focus on weight distribution on the soles of the feet in all the exercises and performed the exercises until symptoms of dizziness and/or unsteadiness were provoked; then to take a short break to let the symptoms subside and then continue with the home exercise program. Examples of the MST home exercises performed in the present study can be viewed at: https://vimeo.com/252733777/c6eef955ad.

A detailed description of the training has previously been published [18] and the exercise program is demonstrated at: https://vimeo.com/album/4948077.

### Control group: wrist stabilization training (WT)

The WT comprised a set of strengthening and coordination exercises for the fractured wrist. The WT exercise set was developed by a physical therapist experienced in the treatment of people with wrist fractures and the exercise set had not been used previously. Post-wrist fracture, muscle weakness and instability of the fractured wrist does often persist and can affect every day function [22]. It was therefore thought likely that this type of exercises could motivate people who had sustained a wrist fracture to participate in the study and adhere to the prescribed exercises. Additionally, we wanted the WT group to receive the same attention from a PT as the MST group, therefore the number and duration of supervised sessions were identical in both groups. The WT was conducted using Inimove training equipment (Inimove ApS, Albertslund, Denmark) in supervised training session. Red elastic resistance bands (TheraBand, Akron. OH), a sponge ball and a ball on a plate were used for the home and supervised exercise sessions. All exercises were performed in a sitting position, in order not to stimulate the control of posture simultaneously (Appendix 2). Examples of the WT exercises performed in the present study can be viewed at: https://vimeo.com/252677340/f0bc12ac32.

The training was supervised by two experienced physiotherapists, who were unaware of the results of the subject’s baseline measurements.

### Outcome measures

All measurements were conducted at baseline and endpoint of the study by the same blinded physiotherapist. The measurements used in the present study are widely accepted, frequently used and validated. The participants were not informed of the assessment results until completion of the training and all measurements. Questionnaires were completed by participants before and after the training.

### Primary outcome measure

#### Posturography

The Sensory Organization Test in the Smart Balance Master (SOT) (Neurocom Inc., Clackamas, OR) was used to measure postural control. The test evaluates the subject’s ability to make effective use of somato-sensory, visual and vestibular inputs and suppress inappropriate sensory information. Composite scores of postural sway from the six different sensory conditions were used for analysis. Age-related normative values for the SOT composite scores have been described, ranging from 64 to 70 [23]. Further description of the SOT and the measuring procedure has previously been published [24]. The test has shown moderate-to-good test–retest reliability in older adults [25] and has been validated among people with vestibular disorders [26].

### Secondary outcome measures

#### Physical function

##### Five Times Sit to Stand Test (FTSTS)

Functional lower limb muscle strength was measured with the FTSTS [27]. Normative performance values for this test across ages have been reported [28]. The test has displayed discriminative validity, concurrent validity and has been shown to be reliable for comparing lower extremity strength in patients from one visit to the next [29].

##### 10-m Walk Test (10MWT)

Gait speed was assessed with the 10MWT. The test was performed at preferred walking speed and repeated at the subject´s fastest speed [30]. This test has shown excellent test–retest reliability for comfortable and fastest gait speeds among healthy adults [31].

#### Sensation in feet

A biothesiometer electronic device (Model EG electronic BioThesiometer, Newbury, OH, USA) that generated a 120-Hz vibration of varying amplitude (in µm) was used to measure vibration perception of the plantar surface of the feet. It was applied to the plantar surface of the caput of the first and fifth metatarsal bones as well as on the center of the plantar surface of the heel. The biothesiometer has shown excellent reliability in testing vibration perception threshold (VPT) within mild-to-moderate neuropathy [32].

The Semmes–Weinstein pressure aesthesiometer (Semmes–Weinstein Monofilaments, San Jose, USA) (SWM) was used to measure tactile sensitivity. The aesthesiometer comprised 20 nylon filaments of equal length, with varying diameters. The filaments were applied to the plantar surface of the same three points as the biothesiometer. Touch threshold is presented as pressure in grams. Normative values of plantar cutaneous SWM threshold have been described and ranges from 0.4 to 4.0 g, depending on testing sites and age [33]. The SWM test is considered the golden standard to screen for loss of protective sensation [34], which is defined as a level of sensory deficit where a patient can sustain an injury without recognizing a trauma [35]. The test has demonstrated acceptable interrater and intra-rater reliability among healthy adults [36].

For statistical analyses mean values of monofilament and biothesiometer were calculated from individual values measured on plantar surface of heel, caput of the first and fifth metatarsal bones, left and right.

#### Vestibular function

##### Head Shake Test

The Head Shake Test was used to assess symmetry of vestibular function. Eye movements were recorded in the supine position with infrared video goggles in place. Based on the recordings, the occurrence of nystagmus and the number of fast eye beats was calculated by a specialist in neurotology. Greater than two or more beats of nystagmus post head shaking was considered positive for vestibular asymmetry [8–10]. The neurotologist was blinded to all recordings both pre- and post-training. The HST, has shown good specificity (82%) but less sensitivity (45%) in pooled analysis [37].

Description of the procedures of all the above tests have previously been described [10].

##### Video-Head Impulse Test (vHIT)

vHIT was used to assess the function of the horizontal semi-circular canals by measuring the eye rotation response to an abrupt head rotation in the plane of the lateral semi-circular canals. The main measure of canal adequacy is the ratio of the eye movement response to the head movement stimulus, i.e., the gain of the vestibulo-ocular reflex (VOR). The measurement of the horizontal VOR by the vHIT has previously been described in detail [38]. The vHIT test was performed with a set of ICS impulse video goggles (GN Otometrics, Taastrup, Denmark), with a camera speed of 250 frames, recording motion of the right eye. Subjects were seated and tested in a well-lit room with an eye-level target at a distance of 1 m. Twenty passive horizontal head turns, both in the right and left directions were performed. The vHIT test has been demonstrated to be a valid clinical tool for testing the function of the horizontal semi-circular canals and simple to use [38, 39].

#### Anthropometrics

Body weight and height were measured and body mass index (BMI) was calculated as kg/m^2^.

#### Questionnaires

##### Activities-Specific Balance Confidence Scale (ABC)

The participants rated their own confidence in 16 activities of daily living on a percentile scale from 0 (no confidence) to 100 (complete confidence) [40]. The scale has demonstrated strong internal-consistency, reliability, and validity when self-administered [41].

##### Dizziness Handicap Inventory Scale (DHI)

Self-perceived handicap resulting from dizziness was assessed with the DHI scale. The scale contains 25 items relating to physical, emotional and functional domains. The range of possible scores on the DHI is 0–100. The higher the score, the greater the level of self-perceived handicap resulting from dizziness [42]. It has been shown to demonstrate change over the course of rehabilitation [43].

Demographics of the participants were obtained using a questionnaire. They were also asked about the level of weekly physical activity, 12 months prior to participation in the study as well as during the training period. They were not instructed to limit their physical activity in any way during participation in the study.

### Data analysis

Statistical analyses were conducted using SPSS for Windows version 24.0 (SPSS, Chicago, IL, USA) and the level of significance was set at *p* < 0.05. Data were checked for normality using the Kolmogorov–Smirnov test. Results are shown as means, standard deviation (SD), median and range. The Mann–Whitney *U* test was used to compare groups at baseline for not normally distributed variables and independent samples *t* test for the normally distributed ones.

Improvements over time were calculated using the Wilcoxon test for the not normally or paired samples *t* test for the normally distributed variables. This was done for each of the training group separately. Effect sizes were calculated according to method described by Cohen for non-parametric variables; effect size (*r*) = *z* divided by square root of total number of observations [44]. Large magnitude of effect is considered to be: *r* ≤ 0.50, medium-sized effects 0.3–0.5 and small effects 0.1–0.3.

Univariate general linear models with statistical adjustment for baseline values, gender and age were used to compare endpoint differences between the two training groups.

## Results

### Baseline

The baseline characteristics of participants are shown in Table 1. The groups were largely comparable, besides vibration sensation, which was significantly poorer in the MST group at baseline (*p* = 0.02).

**Table 1:** Baseline characteristics of the participants categorized by groups

Variable	Wrist training (*n* = 42)			Multi-sensory training (*n* = 38)			*p* value*
	Mean ± SD	Median	Range	Mean ± SD	Median	Range	
Age (years)	60.8 ± 6.7	61	50–75	62.7 ± 7.9	63	50–75	0.38
Sex; females/males (n)	36/6			33/5			
BMI (kg/m^2^)	28.5 ± 6.0	27	19–59	28.4 ± 6.2	28	19–57	0.97
Phys act prev 12 months (h/week)	2 ± 0.8	2	1–3	2 ± 0.6	2.0	1–3	0.36
Falls previous 12 months (n)	2 ± 1.3	1	1–5	2 ± 1.3	1.0	1–6	0.26
Total fractures over lifespan (n)	2 ± 1.2	2	1–6	2 ± 1.6	2.0	1–8	0.52
10MWT comfort speed (m/s)	1.4 ± 0.19	1.4	1.0-1.8	1.4 ± 0.19	1.4	0.7–1.7	0.93
10MWT fast speed (m/s)	1.9 ± 0.3	1.8	1.4–2.7	1.8 ± 0.30	1.8	1.1–2.5	0.42
FTSTS (s)	11.4 ± 2.41	11.2	5.9–15.9	11.70 ± 2.61	11.9	6.8–19.1	0.50
Monofilament (g)	1.6 ± 1.6	1.1	0.3–7.7	1.6 ± 1.1	1.3	0.4–4.3	0.46
Biothesiometer (µm)	2.5 ± 3.8	1.1	0.3–20.0	3.6 ± 4.9	1.8	0.6–25.5	0.02*
Head Shake Test (% positive)^†^	76.2%			89.5%			0.12
vHIT left (gain)	0.9 ± 0.1	0.9	0.8–1.1	0.9 ± 0.1	0.9	0.7–1.2	0.35
vHIT right (gain)	1 ± 0.1	1.0	0.9–1.3	1.0 ± 0.2	1.0	0.8–1.6	0.99
SOT composite (score)	72 ± 7.4	73	50–81	74 ± 7.8	76	52–86	0.11
DHI (score)	9 ± 14.9	1	0–62	13.0 ± 19.6	4	0–66	0.33
ABC (%)	88 ± 13.1	93	43–100	87.0 ± 13.3	90	40–100	0.59

Monofilament and biothesiometer; mean values measured on plantar surface of: heel, caput of the first and fifth metatarsal bones, left and right

Significant values are shown in bold

*BMI*, body mass index, *10MWT* 10 m Walk Test, *FTSTS* Five Times Sit to Stand Test, *vHIT* Video Head Impulse Test, *SOT* Sensory Organization Test, *DHI* The Dizziness Handicap Inventory, *ABC* Activities-Specific Balance Confidence Scale

*Difference between groups according to Mann–Whitney *U* test (not normally distributed variables) and independent samples *t* test (normally distributed variables)

†Positive HST: ≥ 2 fast eye beats

### Drop-out

In the present study, drop-out rate was 10.6% (*n* = 5) in the WT group, and 20.8% (*n* = 10) in the MST group. There were no differences in baseline characteristics between participants who withdrew from the training and those who completed the training in both groups, except that those who stopped in the WT group were older, with a mean age of 70.4 years versus 60.5 years in the MST group.

Participants did not have to provide an explanation for discontinuing training, so data about the reasons for dropping out of the study are limited. However, in the MST group, four dropped out because they felt their balance was fine, three decided to drop out because they did not adhere to the home exercises, two did not arrive for their first training session and one quit because of personal reasons. There was no difference in reporting between the two groups of their exercise adherence.

### Intervention

Table 2 shows within-group mean changes in postural control, foot sensation, vestibular function, perceived dizziness, balance confidence and functional abilities after the intervention. There were significant improvements in both training groups in lower limb muscle strength and SOT. Additionally, there were significant improvements in DHI in the MST group only. The observed effect sizes of significant variables were medium in both groups (MST; SOT: *r* = − 0.34, FTSTS: *r* = − 0.52, DHI: *r* = − 0.30/WT; SOT: *r* = − 0.41, FTSTS: *r* = − 0.34).

**Table 2:** Within-group changes in functional ability, postural control, sensation, perceived dizziness and confidence after the interventions

Outcome measure	Wrist training (*n* = 42)				Multi-sensory training (*n* = 38)			
	*∆*	95% CI		*p* value*	*∆*	95% CI		*p* value*
SOT composite (score)	3.6	1.363	5.813	< 0.01*	4.2	1.495	6.943	< 0.01*
Monofilament (g)	− 0.2	− 0.465	− 0.012	0.14	− 0.1	− 0.360	0.222	0.08
Biothesiometer (µm)	− 0.3	− 0.697	0.133	0.11	− 0.2	− 0.629	0.179	0.38
Head Shake Test positive^a^ (%)	0.0	− 0.195	0.195	1.0	− 15.8	− 0.320	0.005	0.06
vHIT left (gain)	0.01	− 0.012	0.037	0.30	0.0	− 0.011	0.045	0.23
vHIT right (gain)	0.0	− 0.042	0.005	0.15	0.0	− 0.066	0.027	0.41
10MWT comfort speed (m/s)	0.0	− 0.025	0.063	0.39	0.0	− 0.032	0.064	0.51
10MWT fast speed (m/s)	0.0	− 0.037	0.082	0.45	0.1	− 0.002	0.094	0.06
FTSTS (s)	− 1.0	− 1.537	− 0.444	< 0.01*	− 1.5	− 1.964	− 0.996	< 0.001*
DHI (score)	− 2.3	− 5.673	1.088	0.21	− 4.7	− 8.283	− 1.086	0.01*
ABC (score)	0.9	− 1.152	2.935	0.98	2.3	− 0.141	4.743	0.69

Monofilament and biothesiometer; mean values measured on plantar surface of: heel, caput of the first and fifth metatarsal bones, left and right

Significant values are shown in bold

*∆* mean change, *SOT* Sensory Organization Test, *10MWT* 10 m Walk Test, *FTSTS* Five Times Sit to Stand Test, *DHI* The Dizziness Handicap Inventory, *ABC* Activities-Specific Balance Confidence Scale, *vHIT* Video Head Impulse Test

*Baseline − endpoint differences: Wilcoxon non-parametric test and paired samples *t* test

^a^Positive HST: ≥ 2 fast eye beats

Weekly physical activity level during the training period, decreased by 0.02 h/week in the WT group and increased by 0.2 h/week in the MST group, compared to physical activity level 12 months prior to participation in the study. According to Mann–Whitney *U* test, baseline − endpoint differences in physical activity levels between the groups, were not significantly different (*p* = 0.11).

According to linear models, correcting for baseline values, age and gender (Table 3), there was a significant in between group difference in endpoint SOT (MST + 3.1, *p* = 0.01), but not in other outcome variables.

**Table 3:** Endpoint differences between groups in functional ability, sensation, postural control, perceived dizziness and confidence

Outcome measure	*B*	95% CI		*p* value
SOT composite (score)	3.095	0.797	5.393	0.01*
Monofilament (g)	0.134	− 0.158	0.426	0.36
Biothesiometer (µm)	0.250	− 0.139	0.639	0.20
Number fast eye beats	0.135	0.382	0.950	0.88
vHIT left (gain)	0.006	− 0.025	0.037	0.71
vHIT right (gain)	0.002	− 0.037	0.040	0.93
10MWT comfort speed (m/s)	0.002	− 0.056	0.060	0.94
10MWT fast speed (m/s)	0.012	− 0.056	0.081	0.73
FTSTS (s)	− 0.463	− 1.184	0.258	0.20
DHI (score)	− 0.571	− 4.079	2.938	0.75
ABC (score)	1.303	− 1.318	3.924	0.33

Monofilament; mean values measured on plantar surface of: heel, caput of the first and fifth metatarsal bones, left and right

Significant values are shown in bold

*SOT* Sensory Organization Test, *10MWT* 10 m Walk Test, *FTSTS* Five Times Sit to Stand Test, *DHI* The Dizziness Handicap Inventory, *ABC* Activities-Specific Balance Confidence Scale, *vHIT* Video-Head Impulse Test

*Results show MST compared to WT based on univariate general linear models which corrected for base line values, age and gender

### Participants with poor baseline SOT

When looking at within-group changes in participants who had SOT baseline composite scores below age norms, we found that more outcome measures improved in the MST than in the WT group. The observed effect sizes of significant variables in the MST were large (*r* = − 0.64) for all the variables. However, the number of cases was small (MST: *n* = 5, WT: *n* = 8) and statistical power was limited (Table 4). In a separate comparison between groups, using linear regression corrected for baseline values (not shown in a table), we found that the MST had a higher endpoint SOT than the WT group (+ 7.4, *p* = 0.012).

**Table 4:** Within-group changes in functional ability, pressure plantar sensation and postural control after the intervention, among participants with SOT baseline values below age norms

Outcome measures	Wrist training (*n* = 8)				Multi-sensory training (*n* = 5)			
	*∆*	95% CI		*p* value*	*∆*	95% CI		*p* value*
SOT composite (score)	9.5	5	14	0.01*	16.8	12.1	21.5	0.04*
Monofilament (g)	0.1	− 0.2	0.4	0.31	− 0.4	− 1.2	0.4	0.04*
10MWT fast speed (m/s)	0.0	0.0	0.1	0.12	0.1	0.0	0.2	0.04*
FTSTS (s)	− 1.1	− 3.0	0.8	0.16	− 2.9	− 4.7	− 1.1	0.04*

Monofilament; mean values measured on plantar surface of: heel, caput of the first and fifth metatarsal bones, left and right

Significant values are shown in bold

SOT composite age norms (scores): 20–59 years: ≥ 70; 60–69 years: ≥ 68; 70–79 years: ≥ 64

*∆* mean change, *SOT* Sensory Organization Test composite scores, *10MWT* 10 m Walk Test, *FTSTS* Five Times Sit to Stand Test

*Baseline − endpoint differences: Wilcoxon non-parametric test

There were a few outliers in the data set; seven people had 5–6 falls within the previous 12 months, four had DHI scores of > 54 [42] and two had ABC scores of < 50 [45]. The analysis of data was also conducted without the outliers in the analyses, but their removal did not make a difference in the results of the study (data not shown).

## Discussion

The aim of the present study was to investigate whether MST improves postural control, vestibular function, tactile sensation and functional ability among people with fall-related wrist fractures compared to those receiving WT. In a direct comparison, we found that the MST group displayed significantly higher scores on the SOT at the end of the study than WT. Considering within-group changes during the intervention, a significant improvement on DHI was only observed in the MST group but not the WT group. However, significant improvements in both groups were observed for the FTSTS and SOT. According to our results, poor balance control at baseline was associated with a better improvement in postural control during the intervention.

These modest findings were somewhat unexpected, because in a previous pilot study [18], MST resulted in greater improvements in postural control, functional ability and confidence in activities of daily living. This difference in outcomes can possibly be explained by different study populations. The participants in the pilot study were older, between 70 and 92 years, had sustained multiple falls and fractures, most of them had decreased sensation in their lower limbs and they had numerous comorbidities. They were physically weaker, more unstable and less confident during daily activities, as demonstrated by their poorer performance in the different tests and questionnaire (ABC scale) at baseline. The participants in the present study were younger, 50–75 years of age and retrospectively quite healthy and well-functioning. Although, the prevalence of vestibular asymmetry was high (83%) among the wrist fracture participants and 35% of them had reduced plantar sensitivity [33], they were physically active and not complaining of dizziness or unsteadiness as demonstrated by their low DHI and high ABC scores. The balance performance for 84% of them was within normal age range, as measured by the SOT [23]. Walking speed [46] and lower limb functional muscle strength [47] were within normal age-related ranges for healthy individuals among all of the participants and vibration sense [48] was within the normal range for 89% of them. As the participants in the present study were healthy and generally in good physical condition, this might have made it more difficult to achieve improvements with the MST training. Subgroup analysis with wrist fracture participants (WT *n* = 8; MST *n* = 5) with below normal baseline SOT composite scores support this. Reduced postural control at baseline was associated with a better improvement in postural control during the intervention. Additionally, the effects of the intervention among participants with reduced postural control at baseline demonstrated that the MST resulted in significant better outcomes than the WT for these participants. The WT group showed a mean change of 9.5 composite scores on the SOT, which is close to a learning effect (8 scores) due to repeated measurements [49]. However, the MST group exceeded that with mean change of 16.8 scores. Fast walking speed increased by 0.1 m/s post-training among the MST participants, which is regarded clinically meaningful [50], but no change was observed in the WT group. Tactile sensitivity improved as well only in the MST group. Although minimal clinically important differences in tactile sensitivity have not been reported, it has been shown that reduced tactile sensitivity is associated with fall-related wrist fractures [10]. Additionally, a clinically meaningful change of 2.9 s was reached in the FTSTS [51] in the MST group. These results imply that the MST was more effective than the WT among people with reduced postural control. However, as the number of participants with reduced postural control at baseline was very small, these findings need to be confirmed using a larger sample size before firm conclusions can be drawn.

Furthermore, the study protocols in the pilot study and the present study were different which can as well explain the limited improvements observed in the present study. The number of training sessions in the pilot study was dictated by the Icelandic social security reimbursement rules. Each referral included 20 reimbursed sessions, of which two were used for pre- and post-assessments and the remaining 18 for training. The frequency of supervised training sessions in the present study was lower than in the pilot study, consisting of only six supervised sessions and prescribed home exercises. This reduction in supervised sessions was based on clinical experience, where six supervised sessions of the MST and daily home exercises have led to decreased dizziness and improved postural stability among people with unilateral and bilateral peripheral vestibular hypofunction. This approach is as well in line with the clinical practice guidelines from the American Physical Therapy Association for vestibular rehabilitation for peripheral vestibular hypofunction [13]. According to these guidelines, based on expert opinion, persons with chronic unilateral vestibular hypofunction may need supervised sessions once a week for 4–6 weeks, together with daily home exercises.

The drop-out rate in the present study was 11% in the WT group, and 21% in the MST group, which can be considered acceptable in a randomized controlled trial [52].

One variable of interest in this study was asymmetric vestibular function, which has been shown to be associated with falls and wrist fractures [8, 10]. Post-training, there were no significant changes observed on the vHIT in the training groups. The gain of the vestibulo-ocular reflex (VOR) as measured with the vHIT, was within normative values [53] at baseline among the participants so there was most likely a ceiling effect. Conversely, there was a 16% borderline significant (*p* = 0.06) reduction of vestibular asymmetry in the MST group as measured by the Head Shake Test but no change was observed in the group receiving the WT. Previously, Hanson et al. found an 18.5% reduction of vestibular asymmetry after 9 weeks of group sessions two times/week of vestibular rehabilitation among persons post wrist fracture [11]. The reduction in vestibular asymmetry post-training in our study indicates that the MST can positively affect asymmetric vestibular function. This is of importance with regards to fall prevention, as vestibular asymmetry disturbs fall-prevention movements which become smaller or distorted leading to increased danger of falling and imbalance [54]. However, as our findings did not reach statistical significance, no firm conclusions on the effect of the MST on vestibular asymmetry can be drawn from this study.

## Strength and limitations

To the best of our knowledge, no previous studies have investigated the effect of MST among people with fall-related wrist fractures compared to WT. However, it is a limitation that the participants were healthy and well-functioning in accordance with relatively young age thus potentially masking the true potential of the MST.

As in every intervention study, compliance is important. Even though the participants did complete an exercise diary, their adherence to the home exercises cannot be verified.

## Conclusion

MST improves postural control among people who have sustained a fall-related wrist fracture. The results of the study further suggest that the program is more effective for those with balance scores below age-related norms on the sensory organization test.

## Appendix 1: Multi-sensory training

Duration of home exercises: minimum 15 min, 5–7 times each week.

Exercises are performed with bare feet.

Two sets of each exercise are performed. Rest interval between sets, 10–15 s, or until symptoms of dizziness or unsteadiness have subsided.

Throughout all the exercises, focus is on weight distribution on the soles of the feet in order to recognize and control the position and movements of the body.

### Proprioceptive training

Standing. Exercises performed with eyes open and then closed:

1. Weight shift from side to side, 4–6 times in each direction.
2. Weight shift forwards and backwards, 4–6 times in each direction.

Perform exercise (1) and (2) slowly, using smooth corrective motions at the ankles and avoid high-frequency movements at the hips and upper body.

3. Stamping feet on the spot, 7–10 times.

Standing on a balance cushion, foam or trampoline.

- Exercises 1–3 repeated.

Walking (preferred walking speed). 2 min for each exercise.

4. Paying attention to weight distribution on the feet.
5. Stamping feet.
6. Walking on uneven surfaces and surfaces with different textures.

### Vestibular and eye control training

Each of exercises 7–21 are performed until symptoms of dizziness and/or unsteadiness are provoked.

Standing.

7. Eyes kept still during movements of the head in all directions.
8. Moving the head in all directions, eyes following head movements.
9. Moving the head in all directions with eyes closed.
10. Quick movement of the head in all directions with fixed gaze.
11. Quick movement of the head in all directions, eyes following head movements.
12. Quick movement of the head in all directions with eyes closed.

Sitting on a rotational chair.

13. Chair rotated irregularly in both directions with eyes open and closed.

Standing on one foot or on a turning disc.

14. Quick right and left turns, eyes open and closed.

On trampoline.

15. Walking and bouncing.

### Combined proprioceptive and vestibular training

Standing on a balance cushion, foam or trampoline.

- Exercises 7–12.

16. Reaching for an object in different directions.
17. Catching and throwing a ball.
18. Keeping a balloon in the air.

Walking (preferred walking speed).

- Exercises 17–18

19. Moving head in all directions, fixing gaze on surrounding objects.
20. Quick right and left turns.

Sitting on a rotational chair or standing on a turning disc.

21. Chair/disc rotated irregularly in both directions while reading text.

### Fall reaction training

Each of exercises 22–24 are performed 4–6 times in each direction.

Standing.

22. Practicing quick stepping actions in different directions to prevent a fall.
23. Subjects pushed in different directions with and without prior warning.

Walking (preferred walking speed).

24. Subject pushed irregularly in different directions with and without prior warning.

Training method instigated by:

Dr. Ella K. Kristinsdottir, PT, PhD

Bergthora Baldursdottir, PT, MSc

## Appendix 2: Wrist stabilization training

Duration of home exercises: minimum 15 min, 5–7 times each week.

All exercises are performed in the sitting position.

### Exercises using a golf ball on a plate (5 min)

1. Try to keep the golf ball steady in the center of the plate.
2. Try to move the ball slightly without touching the rim of the plate.
3. Move the forearm away from your body while keeping the ball steady in the middle of the cup.

### Exercises using a red elastic band (5 min)

Grab the elastic band using both hands, elbows against the side of the body.

1. Elbows flexed to 90°. Pull the elastic band to the side using the fractured arm resist the pull with the non-fractured arm.
2. One elbow is flexing and the opposite elbow is extending. Pull the elastic band using the fractured forearm by bending the elbow upwards and stabilizing with the non-fractured forearm against the thigh and then resist the forces in the opposite direction.

It is important to maintain the wrist in neutral or slightly extended throughout the exercises.

### Exercises using a sponge ball (5 min)

Squeeze a sponge ball as tightly as possible, count to three and relax, repeat ten times. Rest for a moment (10–15 s) and fully extend the fingers. Repeat the exercise set three times.
